# The Neurotrophic Receptor Tyrosine Kinase in MEC-mPFC Neurons Contributes to Remote Memory Consolidation

**DOI:** 10.1523/JNEUROSCI.2433-21.2022

**Published:** 2022-08-24

**Authors:** Jongryul Hong, Yeonji Jeong, Won Do Heo

**Affiliations:** ^1^Department of Biological Sciences, Korea Advanced Institute of Science and Technology, Daejeon, 34141, Republic of Korea; ^2^Korea Advanced Institute of Science and Technology Institute for the BioCentury, Daejeon, 34141, Republic of Korea

**Keywords:** BDNF cortex, Cre, memory, oligodendrocyte, TrkB

## Abstract

The PFC is thought to be the region where remote memory is recalled. However, the neurotrophic receptors that underlie the remote memory remain largely unknown. Here, we benefited from auto-assembly split Cre to accomplish the neural projection-specific recombinase activity without spontaneous leakage. Deletion of tropomyosin receptor kinase B (TrkB) in neurons projecting from the medial entorhinal cortex to the mPFC displayed reduced remote memory recall from the male mice, but the recent recall was intact. We found that the TrkB deletion attenuates the participation of mPFC cells in the remote fear memory recall. The disruption of remote recall was attributed to reduced reactivation of cells in the mPFC. Notably, TrkB deletion seriously inhibited experience-dependent maturation of oligodendroglia in the PFC, resulting in defects in remote recall that were rescued by clemastine administration. Together, our data suggest that TrkB in intercortical circuits functions in remote memory consolidation.

**SIGNIFICANCE STATEMENT** Retrieving the past experiences or events is essential for the ones to lead life. The investigations performed in the rodent model have disclosed that the systems consolidation of memory accompanying changes of cortical circuits and transcriptome is required for maintaining the memory for a long time. In this study, the split Cre with TrkB^flox/flox^ mice were subjected to discover that TrkB in the neurons plays a role in remote memory consolidation. We evaluated the contextual fear memory and labeled cells, which revealed deletion of TrkB interrupts newborn oligodendrocyte and reactivation of cells in mPFC at remote recall. Our data provide the implication that remote memory is relevant to neurotrophic receptor signaling as well as its influence on non-neuronal cells.

## Introduction

It has been established that recently acquired episodic memory is stored in a cortical-hippocampal trace (Kitamura et al., 2014, 2015; Tanaka et al., 2014; Ryan et al., 2015). Standard theoretical models of systems consolidation suggest that, in rodents, the frontal cortex engages in remote memory recall several weeks after training, although the hippocampus becomes dispensable for remote recall (Frankland and Bontempi, 2005; Tonegawa et al., 2018; Josselyn and Tonegawa, 2020). However, the ensembles of cells that participate in the memory trace, called an engram, remain silent until the original stimuli are given at remote recall in the frontal cortex (Kitamura et al., 2017; DeNardo et al., 2019). It has been shown that changes in synaptic plasticity in hippocampal-cortical networks contribute to formation of remote memory during the time window of memory encoding (Lesburgueres et al., 2011). Inhibition of medial entorhinal cortex (MEC) to mPFC (MEC-mPFC) neurons at the time of memory acquisition hinders generation of engram cells in PFC, suggesting that MEC-mPFC activity is indispensable for permanent storage of memory in the cortex (Kitamura et al., 2017). Considering that this processing takes time, distinct mechanisms might operate throughout remote memory consolidation.

In addition to the role of neurons themselves, non-neuronal cells that participate in remote memory display distinct gene expression signatures (Chen et al., 2020) and arbitrate neuronal communication (Kol et al., 2020). These results demonstrate that the consolidation process requires specific molecular changes in cortical regions. Recent studies have shown that adaptive myelination by oligodendrocytes (OLs) supports learning (McKenzie et al., 2014; Xin and Chan, 2020; Pease-Raissi and Chan, 2021), a process that is also age-related (Hill et al., 2018; Wang et al., 2020) and facilitates remote memory consolidation through the activity-dependent proliferation and differentiation of oligodendroglia in the neocortex (Pan et al., 2020; Steadman et al., 2020). Overall, these observations suggest that storage of memory in the brain requires coordinated communication between neurons and OLs.

Tropomyosin receptor kinase B (TrkB), a member of the neurotrophic receptor tyrosine kinase family, induces tyrosine kinase signaling through binding of the ligand, BDNF, to its extracellular domains. It is well known that BDNF/TrkB signaling is required for synaptic plasticity and memory formation in the hippocampus (Minichiello et al., 1999; Bekinschtein et al., 2007, 2008; Minichiello, 2009; Lu et al., 2011; Lin et al., 2018) as well as the neocortex (Choi et al., 2010; Fritsch et al., 2010; Peters et al., 2010; Choi et al., 2012). These previous reports demonstrate that canonical signal transduction from TrkB is necessary for learning and memory. Interestingly, BDNF/TrkB produces local positive feedback-inducing signal transduction (Harward et al., 2016) and exerts LTP induction at presynapses and postsynapses (Lin et al., 2018). Moreover, BDNF actions can be mediated by paracrine signaling, suggesting that TrkB-dependent BDNF secretion from neurons also induces signaling in non-neuronal cells. In addition to neuronal synapses, BDNF/TrkB signaling in oligodendroglia supports synaptic plasticity and memory formation as a consequence of oligodendroglia maturation (Wong et al., 2013; Fletcher et al., 2018; Geraghty et al., 2019). Thus, these observations suggest that local neurotrophic signaling plays an essential role in learning and memory, although its participation in remote memory has remained in question. In this context, we hypothesized that neurotrophic signaling in neurons supports remote memory consolidation facilitating maturation of OL precursor cells (OPCs) in the mPFC.

Here, we performed MEC-mPFC neurons selective TrkB deletion by expressing Split Cre, which inhibits remote memory recall, not affecting recent memory of the TrkB^Flox/Flox^ mice. We verified that the disruption of the remote recall is because of the decrease of reactivation of cells in the mPFC. Remarkably, we found that TrkB deletion in MEC-mPFC restrains experience-dependent differentiation and maturation of OPCs, which results in suppression of mPFC reactivation. Our discovery demonstrates that TrkB is crucial for remote memory consolidation.

## Materials and Methods

### Experimental model

C57BL/6 mice (8-10 weeks old) were used as WT mice. TrkB^Flox/Flox^ mice were generated by *in vitro* fertilization using sperm obtained from MMRRC (stock #033048-UCD). TrkB^Flox/Flox^ mice were maintained in a C57BL/6 background and used at 7-8 weeks of age. The age of mice for behavior and OPC maturation experiments was strictly maintained. Ai14 mice (The Jackson Laboratory, 007914) were reared in a C57BL/6 background and used at 8-10 weeks of age. TrkB^Flox/Flox^;Ai14 mice were generated by crossing TrkB^Flox/Flox^ and Ai14 mice and were used at 7-9 weeks of age. All mice were maintained on a 12/12 light-dark cycle under the group-housed, and only male mice were used in the experiments due to sex differences in context fear conditioning and generalization (Keiser et al., 2017). Mice subjected to surgery were maintained under the separated cages before the behavior experiment. Food pellet containing doxycycline was supplied depending on the experiments. Mice were randomly assigned to the experiments. Genotyping was performed on all genetically modified mice after the completion of each experimental set. Animal experiments were conducted according to the guidelines of the Institutional Animal Care and Use Committee at the Korea Advanced Institute of Science and Technology.

### DNA vector construction

Split Cre vectors (pAAV-hSyn1::NLS-Split-NCre and pAAV-hSyn1::Split-CCre-NLS-FLAG) were generated using a pAAV-hSyn1::NLS-Cre backbone. Cre was cut between amino acids 69 and 70, yielding NCre and CCre, after which an SV40 NLS sequence and FLAG-tag sequence were added to each split pair. pAAV-CAG::flex-tdT, pAAV-CK0.4::EGFP, and pAAV-Ef1a-DIO-EGFP vectors were purchased from Addgene (#28306, #27226, #37084). The pAAV-RAM::d2tTA-pA-TRE::H2B-mEGFP-pA vector, used for activity-dependent labeling, was generated by introducing the H2B sequence into pAAV-RAM::d2tTA-pA-TRE::EGFP (Addgene #84469). The pAAV-RAM::d2tTA-pA-TRE-hM3D(Gq)-HA vector was generated by introducing the hM3D(Gq) sequence from pAAV-hSyn1-hM3D(Gq) (Addgene #50474) into the above vector, containing a C-terminal HA tag. The backbone vector (Addgene #84469) was a kind gift from Yingxi Lin.

### Adeno-associated virus (AAV) production

All AAVs used in this study were created in our laboratory. AAVs were prepared using the same protocols described in a previous study (Sorensen et al., 2016). Briefly, HEK293T cells were transfected with pAAV vectors, capsid vectors, and helper vector using polyethyleneimine (vector:polyethyleneimine transfection ratio, 1:2.5). Five days after transfection, cells were harvested and treated with nuclease (Millipore, E1014) and lysis chemical (Sigma, 30970), followed by three freeze-thaw cycles. After centrifuging at 13,000 × *g* for 30 min, the supernatants were centrifuged through an iodixanol gradient (OptiPrep, 1114542) at 350,000 × *g* for 1 h (4°C). AAVs in PBS were concentrated using centrifugal filters (Millipore, UFC910096), titrated by qRT-PCR (Takara, 6233), and stored at −80°C until use.

### Stereotaxic surgery and injection

Mice were anesthetized using 200 mg/kg of 2,2,2-tribromoethanol (Sigma, Acros) dissolved in 400-450 µl of PBS. The skull was microdrilled above the target regions, mPFC and MEC. All AAVs were stereotaxically micro-injected at a flow rate of 70nl/min using glass pipettes, which were held in place for 10 min to prevent backflow of injected fluid. For mPFC injections, 0.5 µl of AAVs was injected bilaterally at the following coordinates: AP, 1.7; ML, ±0.2; and DV, 1.55 from the dura. For MEC injections, 0.42 µl of AAVs was injected bilaterally at the following coordinates: AP, −4.85; ML, ±3.75; and DV, 1.65 from the dura. A mixture of AAVs was injected depending on specific experimental protocols, and the final titer of each AAV in mixtures was calculated from original titrations. The serotypes and final injection titers of AAVs used in this experiment were as follows: AAV9-hSyn1::NLS-Split NCre-WPRE-pA, 1 × 10^13^ genome copies (GC)/ml; AAV2-retro-hSyn1::Split CCre-NLS-FLAG-WPRE-pA, 1 × 10^13^ GC/ml; AAV9-CK0.4::EGFP-WPRE-pA, 1 × 10^12^ GC/ml; AAV9-Ef1a::DIO-EGFP-WPRE-pA, 6 × 10^12^ GC/ml; AAV9-CAG::Flex-tdTomato-WPRE-pA, 1 × 10^13^ GC/ml; AAV9-hSyn1::DIO-H2B-mCherry-WPRE-pA, 4 × 10^12^ GC/ml; AAV9-RAM::d2tTA-pA-TRE::H2B-mEGFP-WPRE-pA, 6 × 10^12^ GC/ml; and AAV9-RAM::d2tTA-pA-TRE::hM3D(Gq)-HA-WPRE-pA, 6 × 10^11^ GC/ml. For retrograde tracing of MEC-mPFC neurons, WT mice were injected with 0.5 µl of CTB-Alexa-488 (Thermo Fisher Scientific) at the following coordinates: AP, 1.7; ML, ±0.2; DV, 1.55.

### Contextual fear conditioning

Mice were acclimated to the experimental setting by handling for 4-5 min over 3 d in the behavior room before experiments. For activity-dependent labeling experiments, mice were handled for 2-3 min for 3 d. Contextual fear conditioning was performed in a dimly lit (21 lux), quiet (30 dB) soundproof room. For fear acquisition, 3 times of 2 s foot shocks (0.75 mA) was delivered at 120, 180, and 240 s in Context A (CTX A), after which mice were quickly returned to their home-cage (HC). Context exploration, context only (CTX only), or without experimental actions, HC, on day 1 were performed depending on the experimental procedures (details in each figure). Memory retrieval was performed in either CTX A or B at recent or remote time depending on the experiments (details in each figure). CTX A is an 18.5 cm x 18.5 cm x 32 cm white-transparent acryl box with an electrical grid for shock delivery, and CTX B is a 15 cm diameter x 29 cm high acrylic cylinder with colorful wallpaper and a white flat floor. Each context was cleaned with a sequence of distilled water-70% ethanol-dry wiper after each individual test.

### 2′-Deoxyuridine-5-ethynyl (EdU) administration

A 10 mg/ml of stock solution of EdU was prepared by dissolving 2'-deoxyuridine-5-ethynyl (Carbosynth) in PBS containing 10% absolute ethanol at room temperature. EdU was intraperitoneally administered at a dose of 100 mg/kg to mice in their HC or immediately after fear conditioning.

### Clemastine administration

A 10 mg/ml stock solution of clemastine was prepared by dissolving clemastine fumarate (Sigma) in DMSO (Calbiochem) and stored at −80°C for up to 1 month. The stock solution was diluted to 1 mg/ml in PBS before experiments and intraperitoneally administered to mice at a dose of 10 mg/kg beginning 3 d before fear conditioning and ending 1 d before remote memory retrieval tests. Repetitive injection stress was prevented by performing injections gently and alternating injecting sites between days.

### Clozapine *N*-oxide administration

A 2 mg/ml stock solution of CNO was prepared by dissolving CNO (Tocris) in DMSO and stored at −80°C for up to 1 month. The stock solution was diluted in PBS before experiments and administered intraperitoneally to mice at a dose of 1 mg/kg. Behavior experiments were performed 35-37 min after CNO injection.

### Click chemistry

A Click-iT EdU imaging kit (Alexa-488 and -647) (Thermo Fisher Scientific) was used for detection of EdU incorporation in newly generated cells, as described by the manufacturer. Briefly, fixed sections were blocked by incubating for 1 h with 5% normal donkey serum containing 0.3% Triton X-100. After washing 3 times with wash buffer, the click reaction was performed according to the manufacturer's instructions. After the reaction, sections were washed and reblocked by incubating for 1 h with 10% serum (1:1 goat and donkey) containing 0.3% Triton X-100 in PBS (blocking buffer). Colocalization of EdU and oligodendroglia markers was detected using the same immunostaining procedures.

### Immunostaining

Mice were perfused with 4% PFA in PBS, after which brains were postfixed in 4% PFA in PBS for 24-36 h. Fixed brain tissue was sliced into 45 µm sections using a vibratome (Leica VT1200S). Coronal sectioning was performed first to obtain mPFC sections, after which sagittal sectioning was performed for each subject to obtain the MEC. Slices were blocked and permeabilized by incubating in blocking buffer for 1 h. Donkey serum only was used for PDGFRα labeling owing to antibody-host issues. Slice were incubated for 18-24 h at 4°C with primary antibodies, dissolved in 5% serum containing 0.15% Triton X-100 in PBS. After washing 3 times with 0.3% Triton X-100 in PBS (wash buffer), slices were incubated for 75 min at room temperature with Alexa-488-, -594-, or -647-conjugated secondary antibodies (Thermo Fisher Scientific), diluted 1:1000 in blocking buffer. Sections were then washed 3 times with wash buffer and coverslip-mounted with buffer containing DAPI (Vectashield). The following primary antibodies were obtained from the indicated manufacturers and used at the indicated dilutions: anti-EGFP (Thermo Fisher Scientific, A10262; 1:1000), anti-RFP (Chromotek, 5F8; 1:1000), anti-FLAG (CST, 9A3; 1:1000), anti-PCP4 (Sigma, HPA005792; 1:1000), anti-HA (CST, C29F4, 6E2; 1:1000), anti-Iba1 (Wako, 019-19741; 1:1000), anti-cleaved caspase 3 (CST, 9661; 1:400), anti-c-fos (CST, 9F6; 1:1500), anti-PDGFRα (R&D Systems, AF1062; 1:500), anti-CC1 (Merck, OP80; 1:100), anti-Olig2 (Merck, ab9610; 1:500), anti-phospho S6 (pS6) (CST, 4858; 1:500), and anti-MBP (Merck, ab9348; 1:100).

### Activity-dependent labeling

The following optimal conditions for labeling cells in the mPFC were established for our study. One day before injecting with AAVs expressing RAM::d2tTA-pA-TRE::H2B-mEGFP-WPRE-pA or RAM::d2tTA-pA-TRE::hM3D(Gq)-HA-WPRE-pA (or with each split Cre virus), mice were provided a 1 g/kg Dox diet. After virus injection, mice were allowed to recover for 2 weeks, during which they were provided a 40 mg/kg Dox diet. The Dox diet was withdrawn 24 h (or 36 h) before performing contextual fear conditioning (after mice handling was finished); after fear conditioning was completed, mice were given a 1 g/kg Dox diet for 3-4 h (for H2B-mEGFP expression) or 4-24 h (for hM3D(Gq)-HA expression). The next day, the Dox diet was again changed to 40 mg/kg and then was maintained until the mice were killed. The 40 mg/kg Dox diet, provided *ad libitum* in mouse cages, was replaced every week.

### Statistical analyses

#### Behavior data

For behavior data collection and analysis, freezing motion time was analyzed using FreezeFrame software (Actimetrics). All the freezing video data were collected and calculated under the manufacturer's instruction. For the accuracy, we only analyzed in condition of appropriate AAV expression. Mice were randomly assigned to the fear conditioning. Freezing motion time was estimated blindly.

#### Image data collection and assessment

Images were acquired with Nikon C2 and Nikon A1R HD25 confocal microscopes (Nikon) using 20× and 60× objectives, and image analysis and quantification were performed using NIS-element AR imaging software (Nikon). Data were excluded from analyses in cases where sample conditions were inappropriate or AAV injections in the mouse brain were off target. Data were analyzed under the NIS program tool kits. Slide samples were randomly assigned to estimate expression or colocalization. tdT^+^ and c-fos^+^tdT^+^ cells in the MEC from Ai14 or TrkB^Flox/Flox^;Ai14 mice were quantified by counting the number of labeled cells in the total area of MEC layer 5 from 2 to 4 sections per animal (0.2 mm^2^/387 DAPI^+^). The number of c-fos^+^ cells or percentage of reactivation was calculated as a percentage as follows: number c-fos^+^EGFP^+^ cells/total mEGFP^+^ cells × 100 (%) from 2 sections per animal under denoised conditions (1.57 mm^2^/2525 DAPI^+^). For quantification of the number of oligodendroglia cells, Olig2^+^, EdU^+^Olig2^+^, EdU^+^PDGFRα^+^, EdU^+^PDGFRα^+^Olig2^+^, EDU^+^CC1^+^, and EDU^+^CC1^+^Olig2^+^ cells were counted in 2-4 sections per animal (3.63 mm^2^/5619 DAPI^+^) of the mPFC with area bounded by the corpus callosum border.

#### Statistics

Prism software (GraphPad) was used for statistical assessments of differences between or among samples using unpaired *t* test, paired *t* test, ANOVA, or two-way ANOVA, as appropriate. Statistical information and sample numbers are presented in each figure legend.

### Materials availability

The plasmids and viral vectors generated in this study are available from lead contact on request.

### Data and availability

Any additional information required to reanalyze the data reported in this paper is available from the lead contact on request.

## Results

### Disruption of remote memory retrieval in mice with MEC-mPFC neuron-specific TrkB deletion

**Figure 1.**
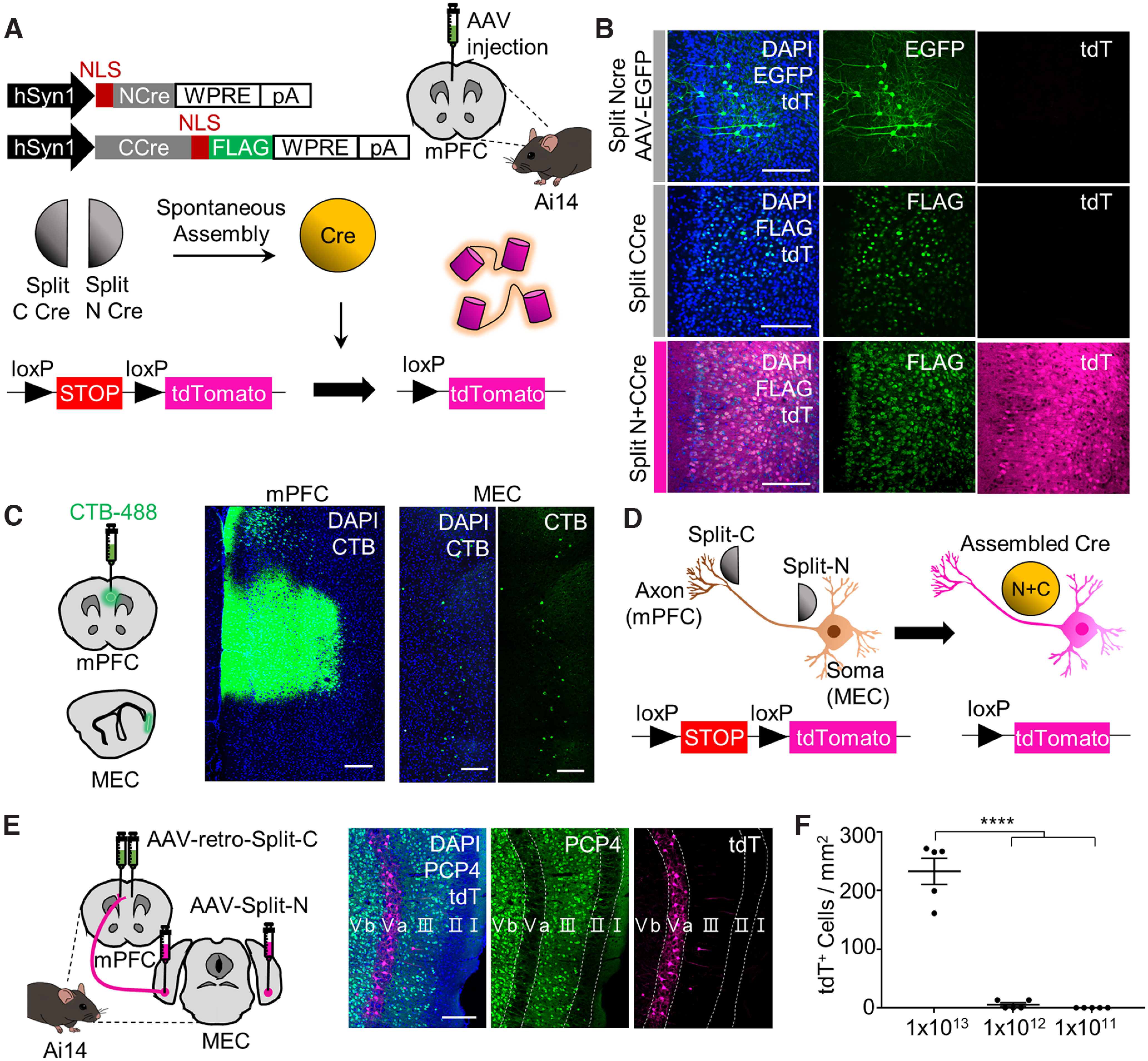
Split Cre gives recombinase activity to MEC-mPFC neurons. ***A***, AAV constructs and injection in Ai14 mice. Split NCre and CCre were delivered via AAV9 and AAV2-retro, respectively. ***B***, Injection of split Cre virus into the mPFC of Ai14 mice. Top, Split NCre injection together with the injection site marker, AAV-CK0.4::EGFP. Middle, Split CCre injection. Bottom, Split NCre and CCre coinjection. ***C***, Left, Illustration of CTB-Alexa-488 injection in the mPFC and brain slicing. Right, Injected mPFC and retrogradely traced neurons in the MEC. ***D***, Illustration of assembled Cre in MEC-mPFC neurons. ***E***, Left, Experimental scheme for labeling MEC-mPFC projecting neurons in Ai14 mice. Right, MEC layer 5 specific tdT expression. ***F***, Viral titration-dependent Cre activity for each split Cre virus pair. Only high titers (1 × 10^13^) produced significant numbers of tdT^+^ cells in the MEC (*n* = 5 for each group). Data in ***F*** are mean ± SEM. ****p < 0.0001. Scale bars, 200 μm.

**Figure 2.**
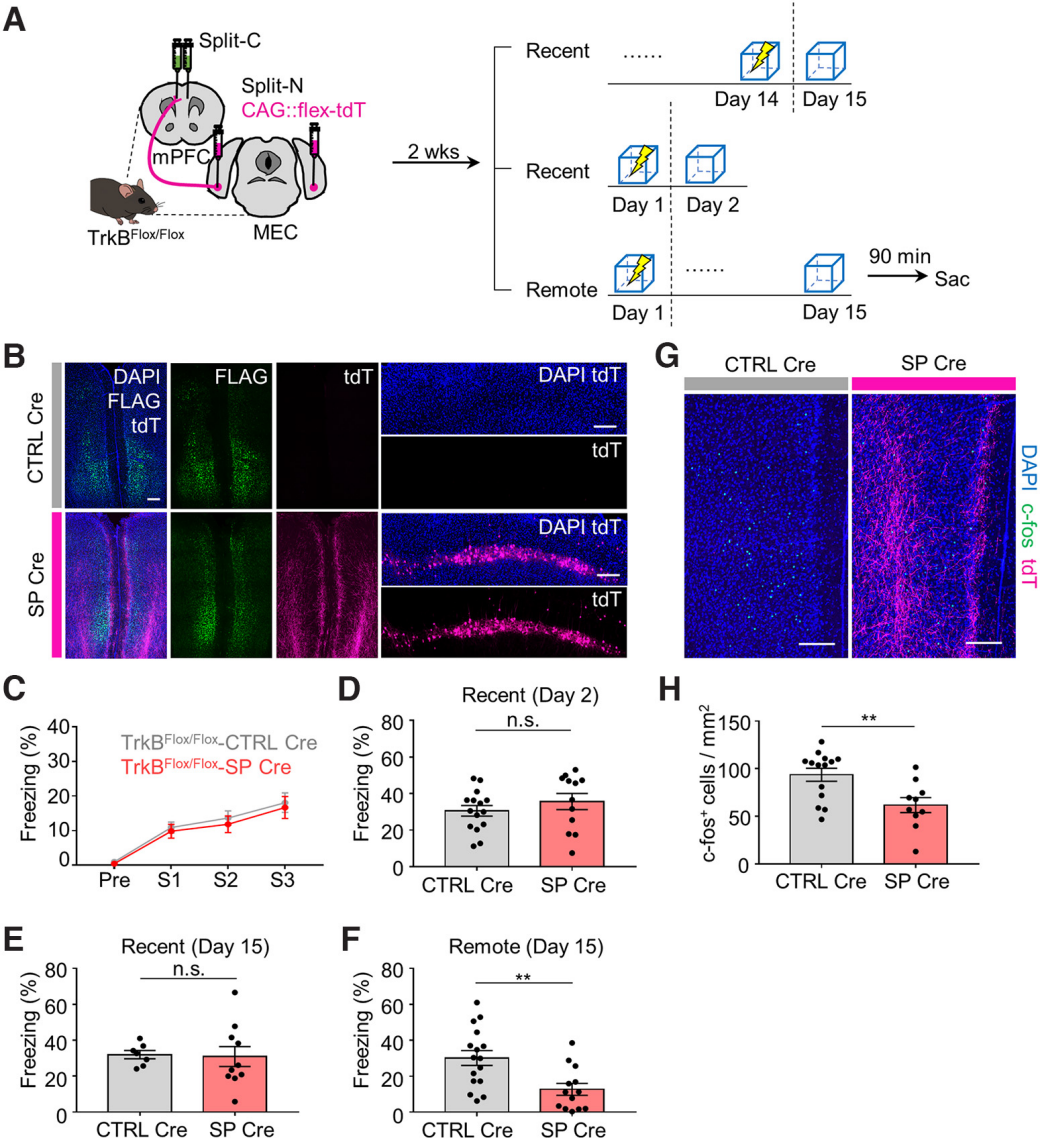
TrkB deletion in MEC-mPFC neurons disrupts remote fear memory recall. ***A***, Experimental procedure for fear conditioning. CFC, Contextual fear conditioning; Sac, sacrifice. ***B***, Representative image showing AAV injection results in ***A***. Left, mPFC. Right, MEC. ***C***, Fear percentage on day 1. TrkB^Flox/Flox^-CTRL Cre (*n* = 31), TrkB^Flox/Flox^-SP Cre (*n* =25) groups were shocked 3 times during conditioning. Pre, pre-shock; S1-3, three time points of electric foot shock. ***D***, Freezing percentage of recent fear memory retrieval (day 2) for CTRL Cre (*n* = 15) and SP Cre (*n* = 12) groups. ***E***, Freezing percentage of recent fear memory retrieval (day 15) for TrkB^Flox/Flox^-CTRL Cre (*n* = 7), TrkB^Flox/Flox^-SP Cre (*n* =10) groups. ***F***, Freezing percentage of remote fear memory retrieval (day 15) for TrkB^Flox/Flox^-CTRL Cre (*n* = 16), TrkB^Flox/Flox^-SP Cre (*n* =13) groups. ***G***, Representative images of mPFC. ***H***, Quantification of c-fos^+^ cells in the mPFC in CTRL Cre (*n* = 14) and SP Cre (*n* =10) groups. Data are mean ± SEM. **p < 0.01. Scale bars, 200 μm.

To test our hypotheses, we first needed to obtain circuit-specific recombinase activity. To eliminate the potential for leakage of Cre recombinase activity when using Flp recombinase with fDIO-Cre for long-term expression (Jung et al., 2019), we adopted Split Cre, which exhibits spontaneous recombinase activity on coexpression of both members of the split pair (Wen et al., 2014). Injection of Split Cre viruses into Ai14 mice resulted in a robust tdTomato (tdT) signal in response to Cre activity (Fig. 1*A*,*B*), but only if coinjected, verifying the previous finding that Split NCre and Split CCre are able to spontaneously assemble in a single cell and function as an active recombinase. We next questioned whether the Split Cre would yield MEC-mPFC-specific Cre activity. We first confirmed that CTB-488 injection in the mPFC yielded a signal in the deep layer of the MEC (Fig. 1*C*). Next, AAV2-retro-hSyn1::Split-CCre-FLAG in the mPFC and AAV9-hSyn1::Split-NCre were injected in the MEC. We observed tdT labeling in MEC layer 5 neurons that project to the mPFC, and confirmed that the Split Cre exhibited recombinase activity in MEC-mPFC neurons (Fig. 1*D*,*E*). However, a high titer of each virus was required to achieve this effect (*n* = 5 mice per group, one-way ANOVA with Sidak's *post hoc* analysis: *F*_(2,12)_ = 104.5, *p* < 0.0001; Fig. 1*F*). For behavior experiments, TrkB^Flox/Flox^ mice were injected in the mPFC with AAV2-retro-hSyn1::Split-CCre-FLAG and in the MEC with AAV9-hSyn1::Split-Ncre with AAV9-CAG::flex-tdT (hereafter, Split CCre with Split NCre, or SP Cre) (Fig. 2*A*). TrkB^Flox/Flox^ mice injected in the mPFC with AAV2-retro-hSyn1::Split-Ccre-FLAG and in the MEC with AAV9-CAG::flex-tdT were used as controls (hereafter, Split Ccre only, or CTRL Cre). Mice in the SP Cre and CTRL Cre groups were then subjected to tests of recent and remote memory. Cells exhibiting tdT^+^ signals in the MEC were observed only in mice of the SP Cre group displaying restored recombinase activity (Fig. 2*B*). Deletion of TrkB in MEC-mPFC neurons did not affect fear acquisition (*n* = 31 and *n* = 25 mice for the CTRL Cre and SP Cre groups, respectively, two-way ANOVA: *F*_interaction(3,162)_ = 0.08676, *p* = 0.9672, *F*_shock (3,162)_ = 58.65, *p* < 0.0001, *F*_Cre(1,54)_ = 0.235, *p* = 0.6298 from Fig. 2*C*) or recent memory retrieval at 2 or 4 weeks after injection of the Split Cre virus (*n* = 15 and *n* = 12 mice for the CTRL Cre and SP Cre groups, respectively, unpaired *t* test: *p* = 0.3289 from Fig. 2*D*; *n* = 7 and *n* = 10 mice for the CTRL Cre and SP Cre groups, respectively, unpaired *t* test: *p* = 0.8834 from Fig. 2*E*). Surprisingly, freezing time remote memory retrieval (day 15) was significantly decreased in the SP Cre group compared with the CTRL Cre group (*n* = 13 and *n* = 16 mice for the SP Cre and CTRL Cre groups, respectively, unpaired *t* test: *p* = 0.0037; Fig. 2*F*). Mice that underwent remote fear memory recall were killed exactly 90 min after completion of the experimental protocol, and mPFC sections were collected and processed for immunostaining. Our immunostaining results showed that there was a significant decrease in c-fos^+^ cells in the mPFC of the SP Cre group compared with the CTRL Cre group. This result indicates that TrkB deletion in MEC-mPFC neurons hinders the activation of mPFC cells under remote memory recall (2 sections per animal from 10 and 14 mice for the SP Cre and CTRL Cre groups, respectively, unpaired *t* test: *p* = 0.0061; Fig. 2*G*,*H*). This finding is in accord with our freezing time results. Collectively, these results indicate that TrkB deletion in MEC-mPFC neurons decreases the retrieval of remote fear memory.

### Reduced reactivation of mPFC cells in mice with MEC-mPFC neuron-specific TrkB deletion

**Figure 3.**
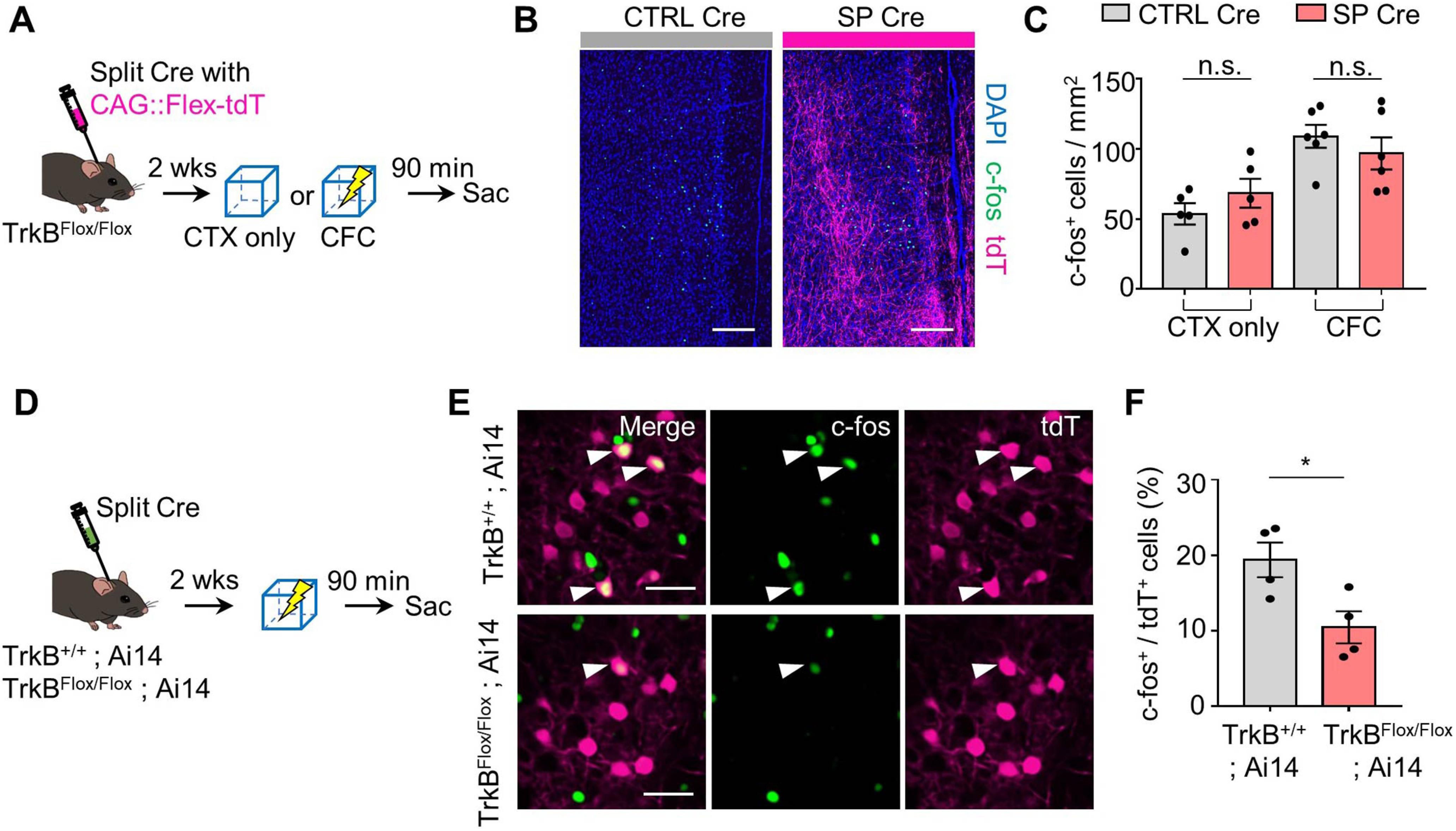
c-fos^+^ cells in mPFC and MEC neurons with fear acquisition. ***A***, Experimental procedure. ***B***, Representative images of the mPFC acquired 90 min after conditioning. ***C***, Quantification of c-fos^+^ cells in the mPFC of SP Cre and CTRL Cre groups under context exploration only (CTX-only) (*n* = 5 each) and CFC (*n* = 6 each). ***D***, Experimental procedure. TrkB^+/+^;Ai14 or TrkB^Flox/Flox^;Ai14 mice were injected with split Cre constructs. ***E***, Representative images of MEC layer 5 from each genotype of mice. White arrows indicate c-fos^+^tdT^+^ cells. ***F***, Quantification of c-fos^+^tdT^+^ (%) in MEC layer 5 from ***E*** (*n* = 4 for each group). Data are mean ± SEM. *p < 0.05. Scale bars: ***B***, 200 μm; ***E***, 50 μm.

**Figure 4.**
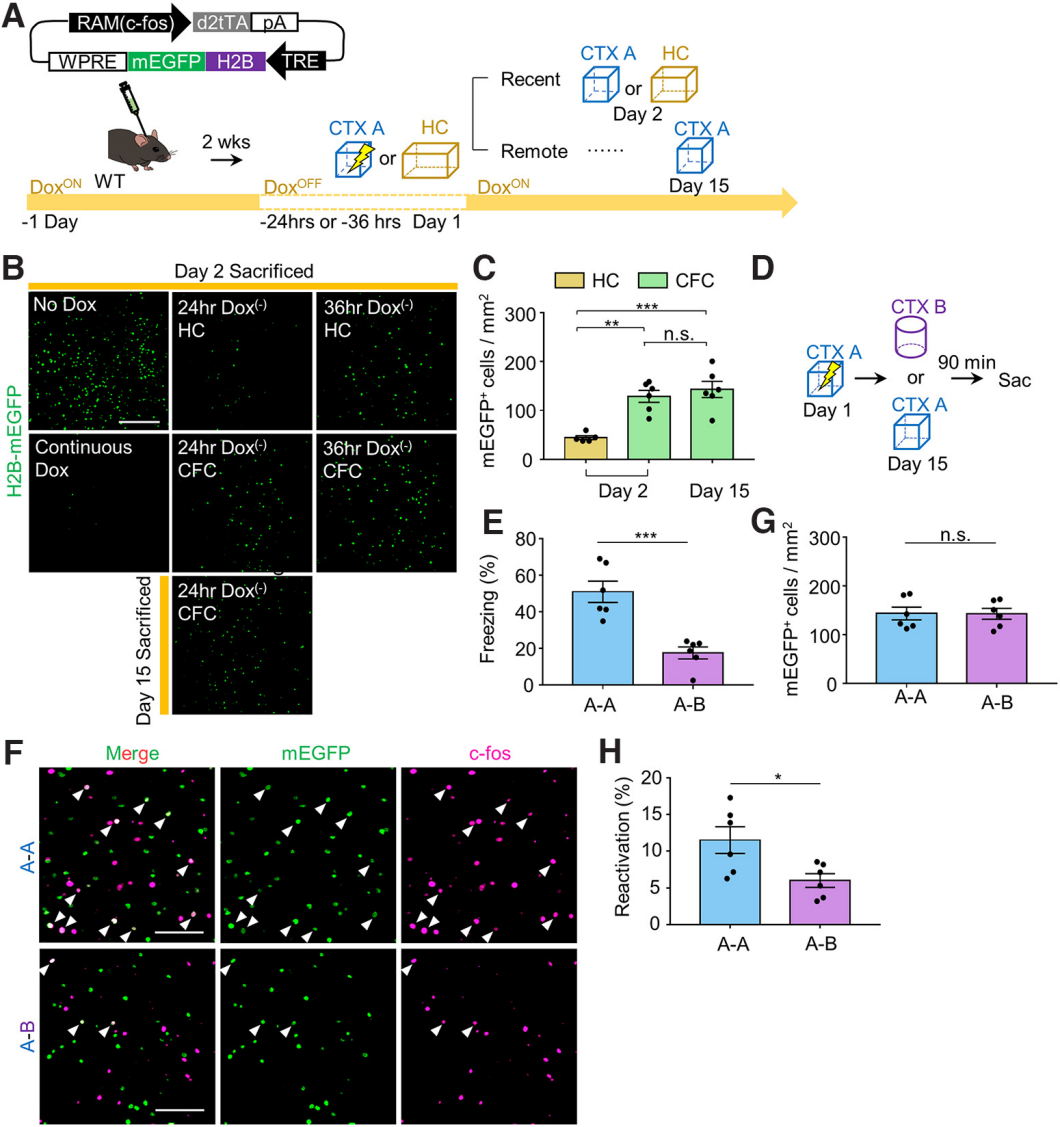
Labeling activity-dependent mPFC cells using the RAM system. ***A***, Experimental procedure for activity-dependent labeling. ***B***, Representative images of H2B-mEGFP expression in the mPFC from ***A***. Less leak was observed for 24 h OFF Dox compared with 36 h OFF Dox for mice in the HC condition. ***C***, Quantification of mEGFP^+^ cells in the mPFC. HC day 2 (*n* = 5), CFC day 2 (*n* = 6), and CFC day15 (*n* = 6) groups. ***D***, Experimental procedure. Labeling procedure is same as that in ***A***, but the retrieval session was performed in CTX A or CTX B at a remote time (day 15). ***E***, Freezing percentage of remote fear retrieval in CTX A (*n* = 6) or CTX B (*n* = 6). ***F***, Representative images of the mPFC from ***D***. White arrows indicate mEGFP^+^c-fos^+^ cells. ***G***, mEGFP^+^ cells in the mPFC from each group (*n* = 6). ***H***, Percentage of reactivation (mEGFP^+^c-fos^+^/mEGFP^+^ cells) of remote retrieval for each group (*n* = 6). Data are mean ± SEM. *p < 0.05. **p < 0.01. ***p < 0.001. Scale bars, 200 μm.

**Figure 5.**
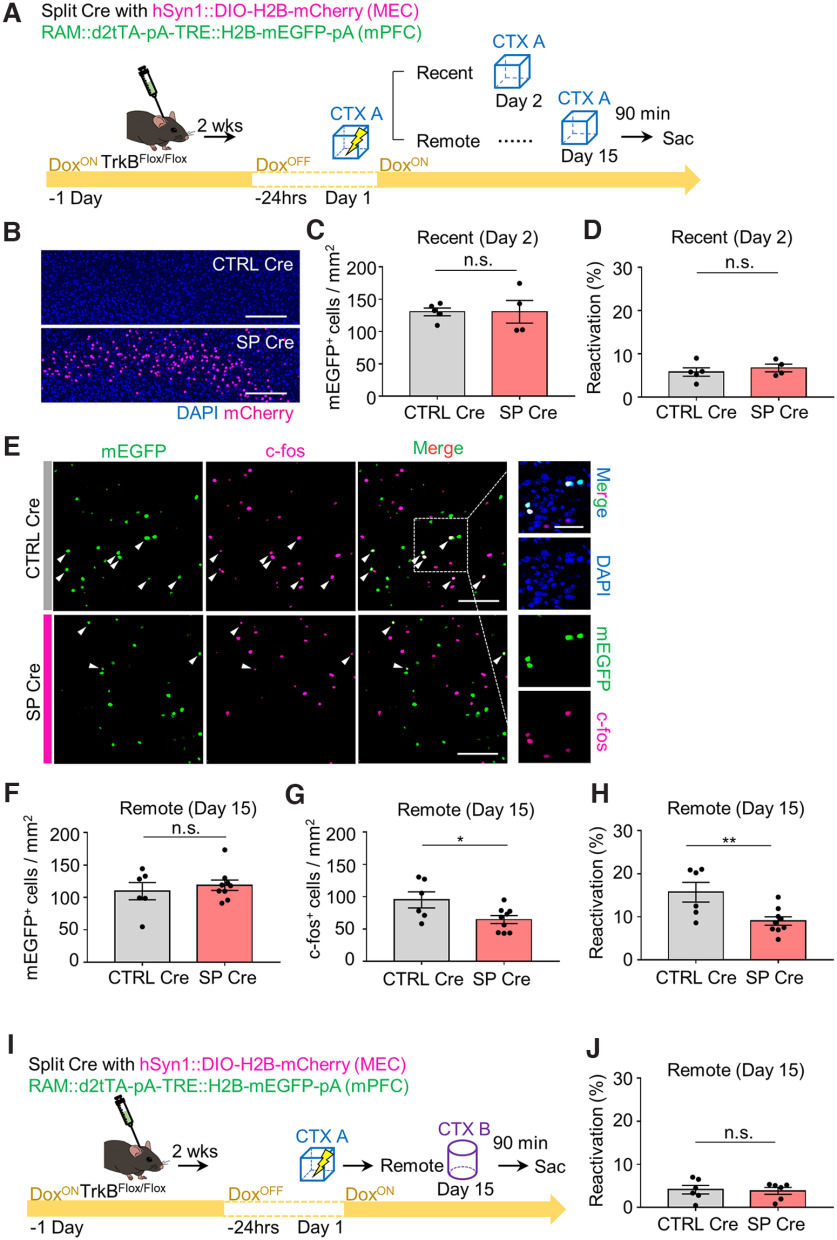
TrkB deletion disrupts reactivation of mPFC cells for remote memory recall. ***A***, Experimental procedure for activity-dependent mPFC cells labeling. ***B***, Representative MEC images of split Cre-dependent H2B-mCherry expression. ***C***, mEGFP^+^ cells from day 2 (*n* = 5 for CTRL Cre, *n* = 4 for SP Cre). ***D***, Percentage of reactivation of recent retrieval from day 2 (*n* = 5 for CTRL Cre, *n* = 4 for SP Cre). ***E***, Representative images showing H2B-mEGFP labeling and immunostaining. Left, mEGFP^+^c-fos^+^ cells (white arrows). Right, Magnified images. ***F***, mEGFP^+^ cells in the mPFC from CTRL Cre (*n* = 6) and SP Cre (*n* = 9) groups. ***G***, c-fos^+^ cells in the mPFC from CTRL Cre (*n* = 6) and SP Cre (*n* = 9) groups. ***H***, Percentage of reactivation (mEGFP^+^c-fos^+^/mEGFP^+^ cells) of remote retrieval in CTRL Cre (*n* = 6) and SP Cre (*n* = 9) groups. ***I***, Experimental procedure for remote retrieval in a different context. ***J***, Percentage of reactivation (*n* = 6 for each group). Data are mean ± SEM. *p < 0.05. **p < 0.01. Scale bars: ***B***, ***E***, left, 200 μm; ***E***, right, 100 μm.

**Figure 6.**
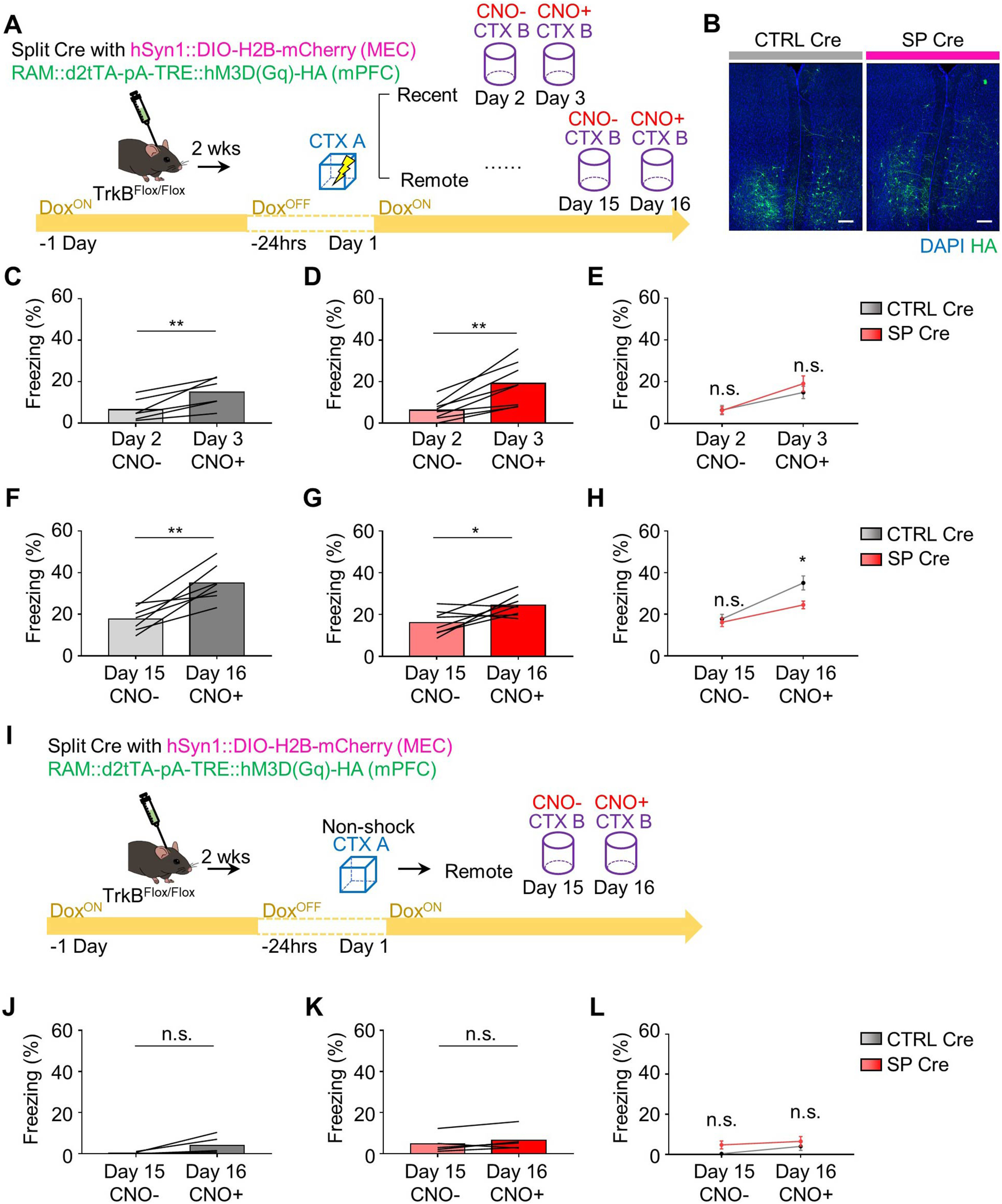
TrkB deletion decreases chemogenetic remote fear memory recall. ***A***, Experimental procedure for chemogenetic activation in CTX B. ***B***, Representative images of hM3D(Gq)-HA expression in the mPFC on day 16. ***C***, Freezing percentage of recent CTX A memory retrieval in CTX B from CTRL Cre (*n* = 6) groups on day 2 and day 3. ***D***, Freezing percentage of recent CTX A memory retrieval in CTX B from SP Cre (*n* = 8) groups on day 2 and day 3. ***E***, Graph comparing results in ***C*** and ***D***. ***F***, Freezing percentage of remote CTX A memory retrieval in CTX B from CTRL Cre (*n* = 7) groups on day 15 and day 16. ***G***, Freezing percentage of remote CTX A memory retrieval from SP Cre (*n* = 8) groups on day 15 and day 16. ***H***, Graph comparing results in ***F*** and ***G***. ***I***, Control experimental procedure. Food shocks were not given to the mice. ***J***, Nonshock CTX A memory retrieval in CTX B from CTRL Cre (*n* = 5) groups on day 15 and day 16. ***K***, Nonshock CTX A memory retrieval in CTX B from Sp Cre (*n* = 5) groups on day 15 and day 16. ***L***, Graph comparing results in ***J*** and ***K***. ***C***, ***D***, ***F***, ***G***, ***J***, ***K***, Data are means and before-after lines. ***E***, ***H***, ***L***, Data are mean ± SEM. *p < 0.05. **p < 0.01. Scale bars, 200 μm.

The decreases in both freezing time at remote recall and the number of c-fos^+^ cells following TrkB deletion in MEC-mPFC neurons raised the question of whether TrkB influences the encoding of fear memory in the mPFC. To answer this question, we injected TrkB^Flox/Flox^ mice with each split Cre virus and killed them exactly 90 min after context exploration-only (CTX only) and CFC experiments (Fig. 3*A*). We found no significant difference in the number of c-fos^+^ cells in the mPFC between the SP Cre and CTRL Cre groups (*n* = 5 mice each for the CTRL Cre and SP Cre groups in CTX only test, *n* = 6 mice each for the CTRL Cre and SP Cre groups in CFC test; for CTX only condition: unpaired *t* test, *p* = 0.2881, and for CFC condition: unpaired *t* test, *p* = 0.4039; Fig. 3*B*,*C*). In the MEC, however, the number of c-fos^+^ neurons projecting to the mPFC was decreased in the SP Cre group of TrkB^Flox/Flox^;Ai14 mice, suggesting that TrkB activity is needed for neural activity in MEC neurons (*n* = 4 mice each for TrkB^Flox/Flox^;Ai14 and TrkB^+/+^;Ai14 groups, unpaired *t* test, *p* = 0.0291; Fig. 3*D-F*). These results led us to hypothesize that TrkB deletion in MEC-mPFC neurons prevents reactivation of cells in the mPFC. To test this, we took advantage of the robust activity marking (RAM) system, in which a tetracycline-controlled transcription activator led by a destabilized domain (d2tTA) is expressed under the control of a minimal c-fos promoter (Sorensen et al., 2016). WT mice were injected with AAV9-RAM::d2tTA-pA-TRE::H2B-mEGFP, after which they were labeled in the mPFC under the 24 h Dox-OFF condition. We found that the mEGFP-labeled signals persisted for 2 weeks (*n* = 5 mice for the day 2 group in the home cage, *n* = 6 mice each for the day 2 and day 15 groups in the CFC test, one-way ANOVA with Sidak's *post hoc* analysis: *F*_(2,14)_ = 16.07, *p* = 0.0002; Fig. 4*A-C*). To determine whether the labeled signals revealed using the RAM system were context-specific, we subjected mice to fear conditioning and retrieval on day 15 in the same context (CTX A) or in a different context (CTX B) (Fig. 4*D*). Labeled mPFC neurons were successfully reactivated without any change in the number of mEGFP^+^ cells (*n* = 6 mice each for the A-A and A-B groups, unpaired *t* test, *p* = 0.0005 from Fig. 4*E*; *n* = 6 mice for each for the A-A and A-B groups, unpaired *t* test, *p* = 0.9699 from Fig. 4*G*; *n* = 6 mice each for the A-A and A-B groups, respectively, unpaired *t* test, *p* = 0.0232 from Fig. 4*H*). Again, we injected TrkB^Flox/Flox^ mice in the mPFC with each split Cre virus together with AAV9-RAM::d2tTA-pA-TRE::H2B-mEGFP, and in the MEC together with AAV9-DIO::H2B-mCherry (Fig. 5*A*). mCherry signals were observed only in the SP Cre group (Fig. 5*B*). No significant difference was observed in the mEGPF^+^ cell population or reactivation percentage at recent recall (*n* = 4 and *n* = 5 mice for the SP Cre and CTRL Cre groups, respectively, unpaired *t* test, *p* = 0.9948 from Fig. 5*C*; *n* = 4 and *n* = 5 mice for the SP Cre and CTRL Cre groups, respectively, unpaired *t* test, *p* = 0.5027 from Fig. 5*D*). In the SP Cre group, however, the number of c-fos^+^ cells was decreased without a change in the mEGFP^+^ population at the remote retrieval. This was accompanied by a reduction of the reactivation percentage (mEGFP^+^c-fos^+^/mEGFP^+^ × 100) (2 sections from *n* = 6 and 9 mice each for the CTRL Cre and SP Cre groups, unpaired *t* test, *p* = 0.5385 from Fig. 5*F*; 2 sections from *n* = 6 and *n* = 9 mice each for the CTRL Cre and SP Cre groups, respectively, unpaired *t* test, *p* = 0.0299 from Fig. 5*G*; 2 sections from *n* = 6 and *n* = 9 mice each for the CTRL Cre and SP Cre groups, respectively, unpaired *t* test, *p* = 0.0094 from Fig. 5*H*). Mice subjected to remote recall in the different context displayed no significant difference of reactivation percentage between the CTRL Cre and SP Cre groups (2 sections from *n* = 6 mice each for the CTRL Cre and SP Cre groups, unpaired *t* test, *p* = 0.8337; Fig. 5*I*,*J*). We next asked whether reduced reactivation of mPFC cells in the Split Cre group is driving the impairments in remote memory recall, we expressed hM3D in MEC-mPFC neurons involved with the CFC response and promoted activation of these cells during recent and remote timepoints in a context (CTX B) that would not normally induce a robust freezing response (Fig. 6*A*). The labeling signal (HA^+^) was observed in both groups (Fig. 6*B*). At recent recall, both groups of mice showed increased fear levels following administration of CNO (day 3) compared with vehicle treatment (day 2) (*n* = 6 mice each for the CNO^–^ and CNO^+^ groups in CTRL Cre mice, paired *t* test, *p* = 0.0081 from Fig. 6*C*; *n* = 8 mice each for the CNO^–^ and CNO^+^ groups in SP Cre mice, paired *t* test, *p* = 0.0021 from Fig. 6*D*). However, we did not observe any significant between-group difference in the fear level at either of the recent retrieval sessions (two-way ANOVA: *F*_interaction (1,24)_ = 0.5954, *p* = 0.4479; *F*_CNO (1,24)_ = 13.85, *p* = 0.0011; *F*_day (1,24)_ = 0.4903, *p* = 0.4905, Sidak's *post hoc* analysis: *p* = 0.9984 for day 2 in the CNO^-^ groups; *p* = 0.5217 for day 3 in the CNO^+^ groups; Fig. 6*E*). These data indicate that the encoding of fear memory in the mPFC is not affected by TrkB deletion. Chemogenetic activation in mice subjected to remote tests was found to increase the fear level (day 16) compared with vehicle treatment (day 15) (*n* = 7 mice each for the CNO^–^ and CNO^+^ groups in CTRL Cre mice, paired *t* test, *p* = 0.0027 from Fig. 6*F*; *n* = 8 mice each for the CNO^–^ and CNO^+^ groups in SP Cre mice, paired *t* test, *p* = 0.0298 from Fig. 6*G*). However, fear levels in mice of the SP Cre group were lower than those in the CTRL Cre group on day 16 (two-way ANOVA: *F*_interaction (1,26)_ = 3.583, *p* = 0.0696; *F*_CNO (1,26)_ = 29.05, *p* < 0.0001; *F*_day (1,30)_ = 6.528, *p* = 0.0168, Sidak's *post hoc* analysis: *p* = 0.8729 for day 15 in CNO^–^ groups; *p* = 0.0082 for day 16 in CNO^+^ groups; Fig. 6*H*). Moreover, we found that chemogenetic activation did not drive significant nonspecific freezing in the TrkB-deleted groups (*n* = 5 mice each for the CNO^–^ and CNO^+^ groups in CTRL Cre mice, paired *t* test, *p* = 0.1149 from Fig. 6*J*; *n* = 5 mice each for the CNO^–^ and CNO^+^ groups in SP Cre mice, paired *t* test, *p* = 0.1738 from Fig. 6*K*; two-way ANOVA: *F*_interaction (1,16)_ = 0.2553, *p* = 0.6202; *F*_CNO (1,16)_ = 2.156, *p* = 0.1614; *F*_day (1,16)_ = 3.473, *p* = 0.0808, Sidak's *post hoc* analysis: *p* = 0.2138 for day 15 in CNO^–^ groups; *p* = 0.5789 for day 16 in CNO^+^ groups from Fig. 6*J*,*L*). Collectively, these results suggest that TrkB deletion in MEC-mPFC neurons disrupts remote memory consolidation in the mPFC.

### Inhibition of experience-dependent generation and maturation of OPCs in mice with MEC-mPFC neuron-specific TrkB deletion

**Figure 7.**
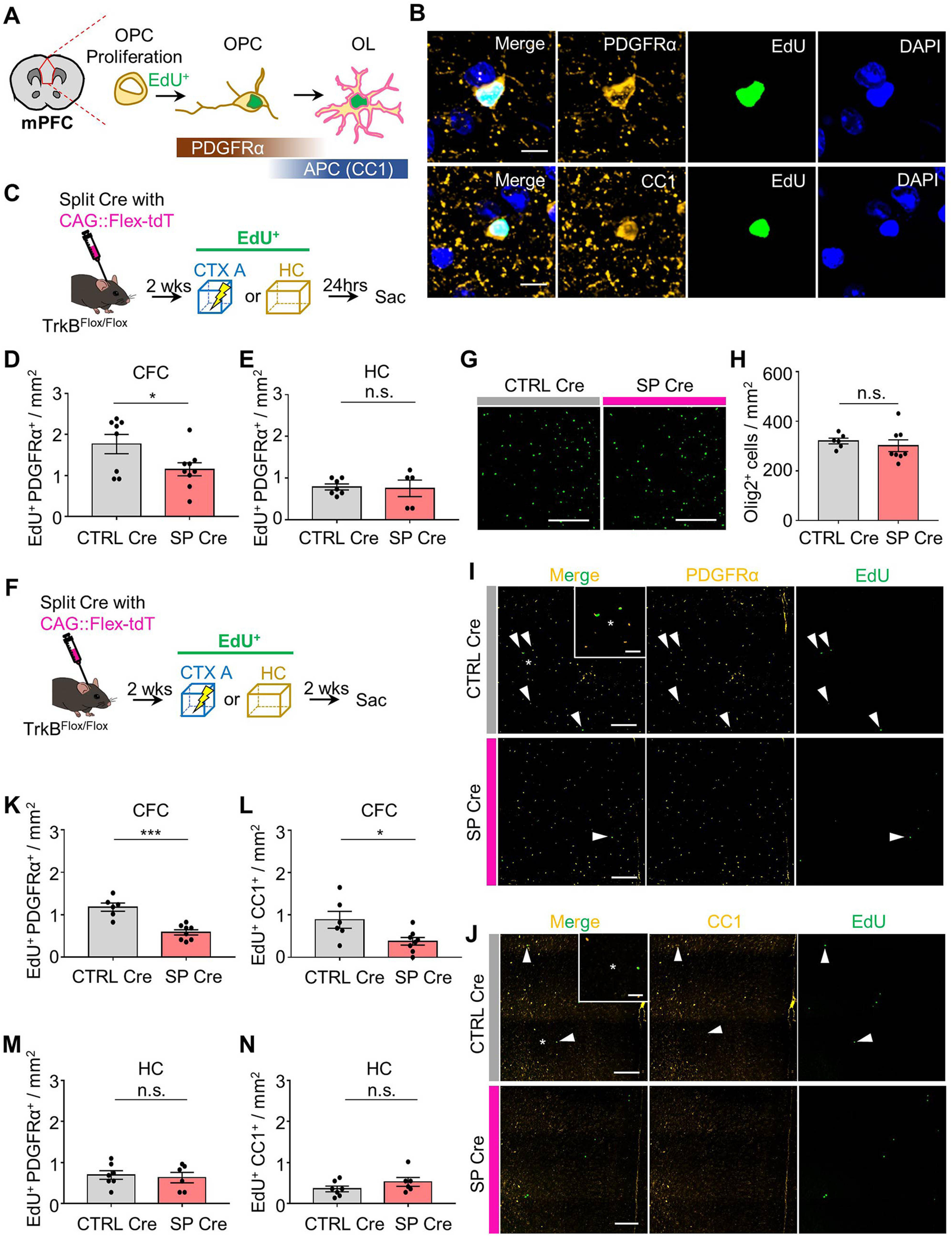
TrkB deletion disrupts experience-dependent oligodendroglia generation and maturation. ***A***, Graphic illustration of the quantification area and EdU labeling of OPCs matured to OLs. ***B***, Representative images of EdU^+^PDGFRα^+^ and EdU^+^CC1^+^ cells in the mPFC at 2 weeks from EdU administration. ***C***, EdU administration at fear conditioning or in the HC and killed after 24 h. ***D***, Quantification of the number of EdU^+^PDGFRα^+^ cells after 24 h from CFC with EdU administration for CTRL Cre (*n* = 8) and SP Cre (*n* = 9) groups. ***E***, Quantification of the number of EdU^+^PDGFRα^+^ cells after 24 h from EdU administration in the HC for CTRL Cre (*n* = 7) and SP Cre (*n* = 5) groups. ***F***, The same protocols with ***C***, but killed after 2 weeks. ***G***, Representative images of Olig2^+^ cells. ***H***, Total number of oligodendroglia in the mPFC for CTRL Cre (*n* = 6) and SP Cre (*n* = 8) groups. ***I***, ***J***, Representative images of mPFC at 2 weeks from EdU administration. EdU^+^PDGFRα^+^ or EdU^+^CC1^+^ cells (white arrows). Magnified images (white asterisks). ***K***, ***L***, Quantification of the number of EdU^+^PDGFRα^+^ (***K***) and EdU^+^CC1^+^ (***L***) cells in CTRL Cre (*n* = 6) and SP Cre (*n* = 8) groups. ***M***, ***N***, Quantification of the number of EdU^+^PDGFRα^+^ (***M***) and EdU^+^CC1^+^ (***N***) cells in CTRL Cre (*n* = 7) and SP Cre (*n* = 6) groups. Data are mean ± SEM. *p < 0.05. ***p < 0.001. Scale bars: ***G***, ***I***, ***J***, 200 μm; ***I***, ***J*** (magnified images), 50 μm; ***B***, 10 μm.

Recent studies showed that a deficit in the maturation of OPCs to OLs constrains remote memory consolidation (Pan et al., 2020; Steadman et al., 2020). We thus asked whether TrkB deletion in MEC-mPFC neurons inhibited OPC generation and maturation. Newly generated OPCs (EdU^+^PDGFRα^+^) and OLs (EdU^+^CC1^+^) were labeled with EdU (Fig. 7*A*,*B*). To determine whether TrkB deletion in MEC-mPFC neurons inhibits experience-dependent OPC generation, we administered TrkB^Flox/Flox^-SP Cre and -CTRL Cre groups with EdU immediately after fear conditioning or in their home cages (controls) (Fig. 7*C*). Surprisingly, the number of newly generated OPCs in mPFC sections from TrkB^Flox/Flox^-SP Cre mice was reduced compared with that in the control group (CTRL Cre) (*n* = 8 and *n* = 9 mice each for the CTRL Cre and SP Cre groups, respectively, unpaired *t* test, *p* = 0.0429; Fig. 7*D*). By comparison, there was no significant difference in the number of newly generated OPCs in the HC mouse group (*n* = 5 and *n* = 7 mice for the SP Cre and CTRL Cre groups, respectively, unpaired *t* test, *p* = 0.8575; Fig. 7*E*). We further asked whether TrkB deletion in MEC-mPFC neurons also constrained OPC maturation. To this end, the TrkB^Flox/Flox^SP Cre and CTRL Cre groups were administered EdU and killed 2 weeks later (Fig. 7*F*). We found that TrkB deletion in MEC-mPFC neurons did not alter the total population of oligodendroglia (Olig2^+^) in the mPFC (*n* = 6 and *n* = 8 mice for the CTRL Cre and SP Cre groups, respectively, unpaired *t* test, *p* = 0.5263; Fig. 7*G*,*H*). Notably, 2 weeks after fear conditioning, the numbers of mature OLs and OPCs were lower in the SP Cre group compared with the control group (*n* = 6 and *n* = 8 mice for the CTRL Cre and SP Cre groups, respectively, unpaired *t* test, *p* = 0.0002 from Fig. 7*K* and *p* = 0.026 from Fig. 7*L*). Again, we did not observe any significant difference in the number of EdU^+^ oligodendroglia in the HC groups (*n* = 6 and *n* = 7 mice for the SP Cre and CTRL Cre groups, respectively, unpaired *t* test, *p* = 0.7066 from Fig. 7*M* and *p* = 0.1971 from Fig. 7*N*). Overall, these results demonstrate that TrkB deletion in MEC-mPFC neurons inhibits OPC maturation and experience-dependent OPC generation in the mPFC.

### Recovery of remote memory recall by chemical restoration of OPC maturation

**Figure 8.**
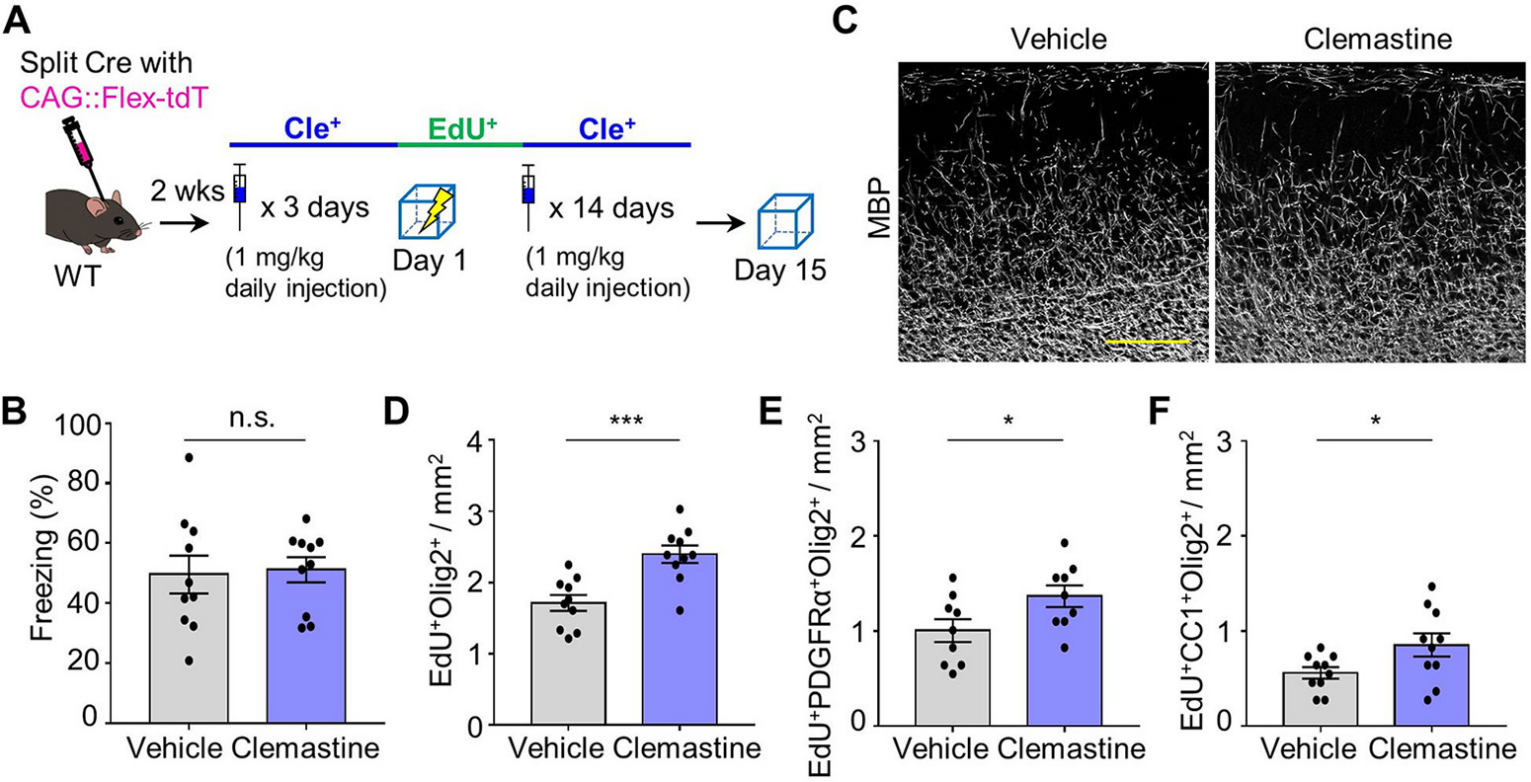
Effect of administration of clemastine on remote memory and oligodendroglia generation. ***A***, Experimental procedure for clemastine administration in WT mice. ***B***, Freezing percentage of remote fear memory retrieval in vehicle and clemastine treatment groups (*n* = 10 for each group). ***C***, Representative images of the myelin basic protein (MBP) in mPFC from vehicle or clemastine treatment groups. ***D–F***, Quantification of EdU^+^Olig2^+^ (***D***), EdU^+^PDGFRα^+^Olig2^+^ (***E***), and EdU^+^CC1^+^Olig2^+^ (***F***) cells in the mPFC from vehicle or clemastine treatment groups (*n* = 10 for each group for ***D–F***). Data are mean ± SEM. *p < 0.05. ***p < 0.001. Scale bars, 200 μm.

**Figure 9.**
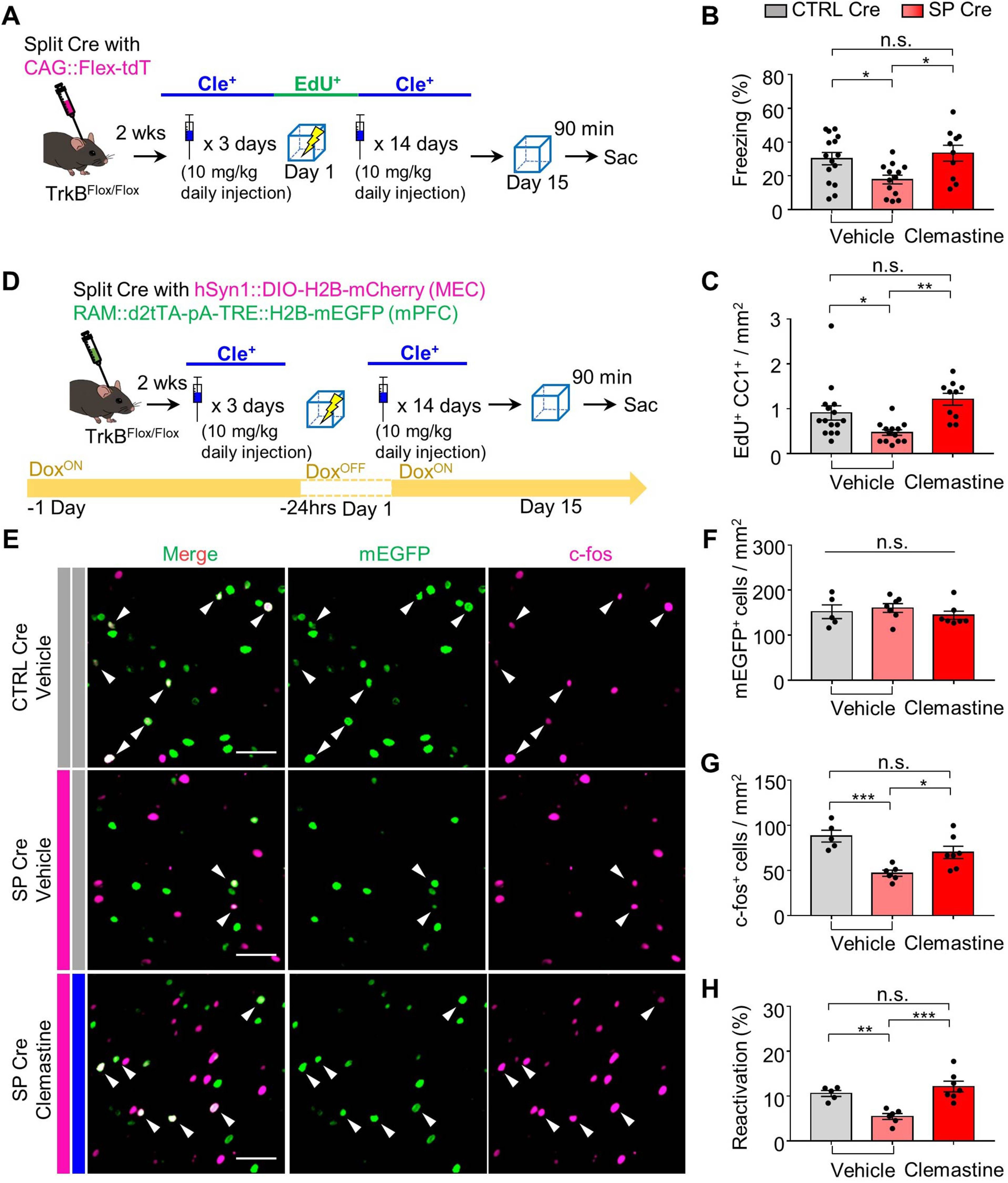
Clemastine restored disrupted remote memory recall through maturation of OPCs. ***A***, Experimental procedure for clemastine injection together with EdU labeling in TrkB deleted mice. ***B***, Freezing percentage of remote fear memory retrieval in CTRL Cre (Veh^+^) (*n* = 15), SP Cre (Veh^+^) (*n* = 13), and SP Cre (Cle^+^) (*n* = 10) groups. ***C***, Quantification of the number of EdU^+^APC^+^ cells in CTRL Cre (Veh^+^) (*n* = 15), SP Cre (Veh^+^) (*n* = 13), and SP Cre (Cle^+^) (*n* = 10) groups. ***D***, Experimental procedure for clemastine injection together with RAM system labeling. ***E***, Representative mPFC images of ***D***. ***F***, mEGFP^+^ cells in the mPFC of CTRL Cre (Veh^+^) (*n* = 5), SP Cre (Veh^+^) (*n* = 7), and SP Cre (Cle^+^) (*n* = 7) groups. ***G***, c-fos^+^ cells in the mPFC of CTRL Cre (Veh^+^) (*n* = 5), SP Cre (Veh^+^) (*n* = 7), and SP Cre (Cle^+^) (*n* = 7) groups. ***H***, Percentage of reactivation (mEGFP^+^c-fos^+^/mEGFP^+^) of remote memory recall in CTRL Cre (Veh^+^) (*n* = 5), SP Cre (Veh^+^) (*n* = 7), and SP Cre (Cle^+^) (*n* = 7). Data are mean ± SEM. *p < 0.05. **p < 0.01. ***p < 0.001. Scale bars, 50 μm.

**Figure 10.**
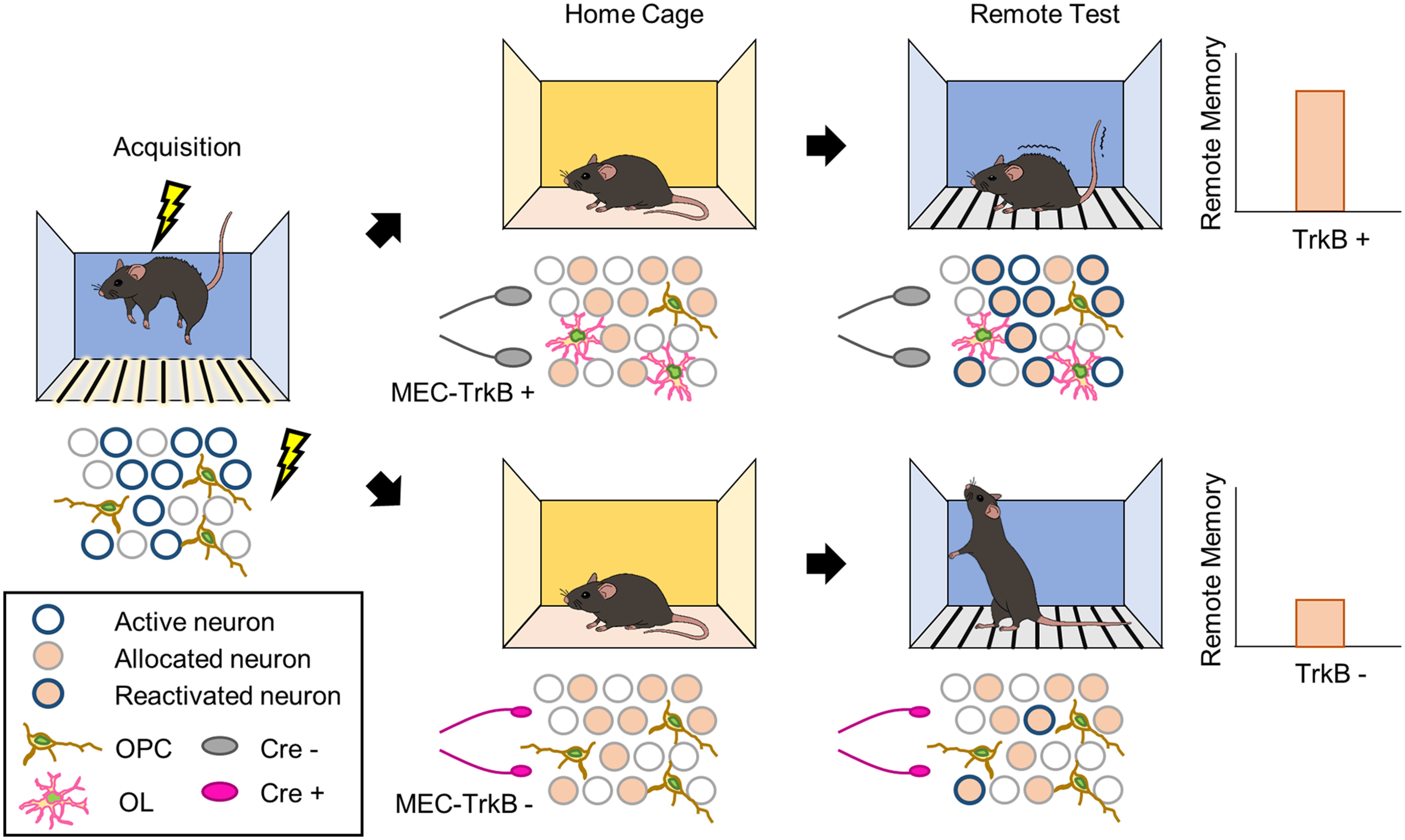
Graphic summary of TrkB in MEC-mPFC contributes to remote memory consolidation through OPC maturation. TrkB in MEC neurons projecting to the mPFC contributes to maturation of OPCs, thereby consolidating remote memory.

To investigate whether the disruption in remote memory retrieval is caused by inhibition of OPC maturation resulting from TrkB deletion in MEC-mPFC neurons, we administered clemastine, which promotes OPC maturation (Mei et al., 2014; Liu et al., 2016). Treatment of WT mice with clemastine from 3 d before fear conditioning to 1 d before remote memory testing did not enhance remote memory (*n* = 10 mice each for vehicle and clemastine groups, unpaired *t* test, *p* = 0.8356; Fig. 8*A*,*B*) or cause an overt increase of myelination in deep layers (Fig. 8*C*). However, it did significantly increase OPC generation and maturation in the mPFC (*n* = 10 mice each for vehicle and clemastine groups, unpaired *t* test: *p* = 0.0006 from Fig. 8*D*, *p* = 0.0439 from Fig. 8*E*, and *p* = 0.0452 from Fig. 8*F*). We next examined whether clemastine treatment could rescue the disruption in remote fear memory recall. To this end, the TrkB^Flox/Flox^-SP Cre and -CTRL Cre groups were administered clemastine (or vehicle) and fear conditioning was performed (Fig. 9*A*). Clemastine treatment reversed the disruption of remote memory recall in association with an increase in OLs in the TrkB-deleted group (*n* = 15 for vehicle group in CTRL Cre mice, *n* = 13 for vehicle group in SP Cre mice, *n* = 10 for clemastine group in SP Cre, one-way ANOVA with Sidak's *post hoc* test: *F*_(2,35)_ = 4.984, *p* = 0.0128 from Fig. 9*B*; *n* = 15 for vehicle group in CTRL Cre mice, *n* = 13 for vehicle group in SP Cre mice, *n* = 10 for clemastine group in SP Cre mice, one-way ANOVA with Sidak's *post hoc* test: *F*_(2,35)_ = 7.458, *p* = 0.002 from Fig. 9*C*). Finally, to determine whether the recovery of remote memory recall resulted from reactivation of cells in the mPFC through OPC maturation, we administered clemastine (or vehicle) to mice injected with split Cre (or control virus) and the RAM system (Fig. 9*D*). Clemastine injection rescued the number of c-fos^+^ cells without affecting the number of mEGFP^+^ cells (*n* = 5 for vehicle group in CTRL Cre mice, *n* = 7 each for vehicle and clemastine groups in SP Cre, one-way ANOVA with Sidak's *post hoc* test: *F*_(2,15)_ = 0.606, *p* = 0.5576 from Fig. 9*F*; *n* = 5 for vehicle group in CTRL Cre mice, *n* = 7 each for both vehicle and clemastine group in SP-Cre mice, one-way ANOVA with Sidak's *post hoc* test: *F*_(2,15)_ = 11.13, *p* = 0.0011 from Fig. 9*G*). This restored the reactivation (*n* = 5 for vehicle group in CTRL-Cre mice, *n* = 7 for the vehicle and clemastine groups in SP-Cre mice, one-way ANOVA with Sidak's *post hoc* test: *F*_(2,15)_ = 14.09, *p* = 0.0004; Fig. 9*H*). Together, these results demonstrate that TrkB deletion in MEC-mPFC neurons inhibits experience-dependent OPC maturation in the mPFC and thereby reduces remote memory recall (Fig. 10).

## Discussion

Memory goes through consolidation steps to become a long-lasting memory that can be recalled throughout an entire lifetime. The hippocampal-cortical network supports memory ensembles in the PFC where cells are activated for remote memory (Guo et al., 2018). The superficial layers of the entorhinal cortex to the hippocampus serve memory acquisition, which is also stored in the frontal cortex through the entorhinal-prefrontal network (Takehara-Nishiuchi, 2014; Witter et al., 2017). Prominent models of remote memory consolidation suggest a role for continuing changes in intercortical connections (Tonegawa et al., 2018; DeNardo et al., 2019; de Sousa et al., 2019), consistent with the idea that synaptic and genetic changes in the frontal cortex may underlie consolidation of remote memory (Bero et al., 2014; Chen et al., 2020). Although there are emerging studies of neural circuits for remote memory consolidation, there is a lack of studies investigating how neurotrophic receptors serve remote memory.

Using circuit-specific delivery of an AAV-based, leak-free split Cre system, we achieved TrkB deletion in MEC-mPFC neurons in TrkB^flox/flox^ mice. MEC-mPFC neuron-specific deletion of TrkB reduced fear memory recall, specifically affecting remote memory and not recent memory. The reduced number of c-fos^+^ cells in the mPFC of mice with MEC-mPFC neuron-specific deletion of TrkB indicates that cells in the mPFC do not promptly participate when mice are exposed to the same context during the remote memory recall session. This decrease resulted in reduced reactivation of cells that participated in the encoding stage. Using activity-dependent labeling in the mPFC, we were able to observe that disruption of remote memory recall in MEC-mPFC TrkB-deleted mice is attributable to suppressed reactivation of cells in the mPFC. Furthermore, chemogenetic activation showed that memory encoding cells in the mPFC are not fully available for remote memory recall in MEC-mPFC TrkB-deleted mice.

However, the encoding process in the mPFC of MEC-mPFC TrkB-deleted mice remained intact, a conclusion supported by several findings. First, there was no significant difference in the number of c-fos^+^ cells in the mPFC between MEC-mPFC TrkB-deleted and control mice at 90 min after contextual fear memory acquisition. There was also no significant difference in the number of labeled cells determined using the activity-dependent labeling system (*RAM/TetO*::H2B-mEGFP), suggesting that participation of the mPFC in memory acquisition is not affected by TrkB deletion in MEC-mPFC neurons. Finally, chemogenetic activation of labeled cells in the mPFC enabled recall of recent memory, but the roles of these cells in remote recall became faint in TrkB-deleted mice. These results suggest that TrkB deletion in MEC-mPFC neurons may cause deterioration in the availability of mPFC cells for formation of a fear memory trace, despite successful encoding at memory acquisition. Collectively, our data suggest that the memory consolidation hinges on neurotrophic signaling in MEC neurons.

Notably, studies have established that proliferation and differentiation of OPCs are required for remote memory (Pan et al., 2020; Steadman et al., 2020), demonstrating that new myelination in axon-projecting interbrain regions is essential for cortical consolidation. Adaptive myelination is induced by neural activity (Wake et al., 2011; McKenzie et al., 2014), dynamically regulated (Yang et al., 2020), and continues until death (Hill et al., 2018). Thus, storing and maintaining memory in cortical networks require adaptation and preservation of myelination for communication with neuronal ensembles (Bonetto et al., 2021).

Our data show that TrkB deletion in MEC-mPFC neurons disrupted the natural properties of OPCs in the mPFC. Interestingly, MEC-mPFC TrkB deletion significantly reduced experience-dependent proliferation and differentiation of oligodendroglia as well as remote memory recall. Moreover, these phenomena were restored by daily administration of clemastine. These results show that TrkB in MEC-mPFC neurons plays a critical role in experience-dependent OPC maturation in the mPFC, but additional research is necessary to elucidate the detailed mechanisms by which neuronal TrkB orchestrates the neurons and non-neuronal cells responsible for remote memory consolidation. One conceivable mechanism that has been proposed is that TrkB-mediated activity-dependent release of the neurotrophic factor, BDNF, from axon terminals (Fletcher et al., 2018; Geraghty et al., 2019; Woo et al., 2019) evokes the consolidation stage of OPC maturation, a possibility that remains to be investigated.
